# An audit on pneumococcal vaccination rates among adult patients with asthma and chronic obstructive pulmonary disease (COPD) in a local polyclinic

**DOI:** 10.1186/2047-2994-4-S1-P108

**Published:** 2015-06-16

**Authors:** QCT Chang

**Affiliations:** 1 Family Medicine, National Healthcare Group Polyclinics, Singapore

## Introduction

Pneumococcal disease is a preventable cause of morbidity and mortality. It can lead to pneumonia, bacteremia and meningitis. S. pneumonia infection is the most common cause of community acquired pneumonia worldwide^1^. The common serotypes isolated were all covered by the pneumococcal vaccine. Smokers, patients with heart disease, asthma, COPD, diabetes mellitus, alcoholism, liver cirrhosis as well as the immunocompromised are at a greater risk and should be vaccinated. All adults aged 65 years and older should also get the adult Pneumococcal vaccine as long as it has been 5 years since the previous dose and they have no contraindications^2^.

## Objectives

To increase uptake of adult Pneumococcal vaccination rates in patients with Asthma and COPD in Bukit Batok Polyclinic from September to November 2013 to at least 10%.

## Methods

All patient visits coded with the Diagnoses “Asthma” and/or “COPD” in the months of May-November 2013 were retrieved from the clinic database with the help of our clinic operations executive and IT support staff. Those who were administered the adult Pneumococcal vaccine (PPSV23) vaccine in these months were identified. Patients who were less than 19 years old, or were already previously vaccinated were excluded. A root cause analysis showed multiple physician, system and patient factors. Appropriate interventions were put in place and the vaccination rates were reassessed.

## Results

The results showed an improvement in uptake rates with the highest rate of 5.99% in October 2013.

## Conclusion

Physician recommendation and increasing patient awareness of the vaccine were effective in increasing uptake rates. Doctors should target suitable patients for vaccination in every doctor-patient contact, dispel misconceptions, offer information and provide adequate counselling about the risks and benefits of the vaccine. Electronic prompting to remind physicians to offer vaccinations and clinical audit of immunisation rates is likely to be an effective way of improving and monitoring vaccine uptake rates.

## Disclosure of interest

None declared.
